# Real-world adherence, healthcare resource utilization, and costs following a transition to aripiprazole once every 2 months among patients diagnosed with bipolar I disorder in the United States

**DOI:** 10.3389/fpsyt.2026.1904737

**Published:** 2026-09-08

**Authors:** Soma S. Nag, Annette Urganus, Karimah S. Bell Lynum, Norman Atkins, Onur Baser, Katarzyna Rodchenko, Shivanshu Awasthi, Kristine Harrsen

**Affiliations:** 1 Otsuka Pharmaceutical Development & Commercialization Inc., Princeton, NJ, United States; 2 Lundbeck LLC, Deerfield, IL, United States; 3 Graduate School of Public Health, City University of New York (CUNY), New York, NY, United States; 4 Columbia Data Analytics, New York, NY, United States; 5 Department of Economics, Boğaziçi University, Istanbul, Türkiye; 6 H. Lundbeck A/S, Valby, Denmark

**Keywords:** adherence - compliance - persistence, aripiprazole monohydrate, bipolar I disorder, healthcare resource utilization (HCRU), long-acting injectable (LAI), real-world evidence (RWE), retrospective cohort study

## Abstract

**Introduction:**

Transitioning from oral to long-acting injectable (LAI) antipsychotics may improve adherence and reduce healthcare resource utilization (HCRU) in patients living with bipolar I disorder (BP-I), with potentially greater improvements with LAIs with dosing intervals longer than 1 month. Aripiprazole monohydrate is available as LAI formulations administered once-monthly [aripiprazole once-monthly (AOM)] or once every 2 months (aripiprazole 2-month ready-to-use [Ari 2MRTU]). This pre-post analysis described adherence, HCRU, and costs over 6 months in adults diagnosed with BP-I who transitioned from oral antipsychotics or AOM to Ari 2MRTU.

**Methods:**

This retrospective, noninterventional study used Kythera Labs United States closed claims to identify adults diagnosed with BP-I, commercially or Medicaid-insured, and who transitioned from oral antipsychotics or AOM to Ari 2MRTU from April 2023–September 2024 (index date = Ari 2MRTU transition). Continuous enrollment for 12 months pre- and 6 months post-index was required. Study outcomes were compared 6 months prior to and following the index date.

**Results:**

Among those who transitioned from oral antipsychotics to Ari 2MRTU (n = 371),mean proportion of days covered (PDC) increased from 0.60 to 0.76 (26.2% relative increase), mean medication possession ratio (MPR) increased from 0.67 to 0.85 (26.8% relative increase), and proportion of patients with MPR ≥ 0.8 increased from 41.2% to 72.0% (74.5% relative increase) (all *P* < 0.0001). Notable reductions in all-cause inpatient visits and length of stay (LOS) were observed, along with reductions in BP-I–related LOS and emergency department (ED) visits. All-cause inpatient, ED, and total medical (inpatient, outpatient, ED) costs declined. Among those who transitioned from AOM to Ari 2MRTU (n = 1,096), mean PDC increased from 0.56 to 0.81 (45.3% relative increase), mean MPR increased from 0.62 to 0.89 (43.7% relative increase), and proportion of patients with MPR ≥ 0.8 increased from 51.2% to 79.0%) (54.4% relative increase) (all *P* < 0.0001). Inpatient visits (all-cause, BP-I–related), and all-cause LOS decreased, along with all-cause ED and total medical costs.

**Discussion:**

Transitioning to Ari 2MRTU was associated with improved treatment adherence and reduced selected HCRU measures and total medical costs, supporting its value as a treatment option for BP-I.

## 1 Introduction

Bipolar I disorder (BP-I) is a lifelong psychiatric condition characterized by at least one manic episode, which may be preceded or followed by hypomanic or depressive episodes (1). The lifetime prevalence of BP-I among adults is estimated to be 0.6–1.1% globally (2, 3) and 2.1% in the United States (US) (4). Individuals living with BP-I experience reduced quality of life, impaired functioning, and cognitive impairment compared with healthy individuals or those without BP-I or other mental health disorders (5–7). In the US, BP-I is associated with significant direct and indirect healthcare expenditures relative to the general population, driven largely by caregiving, direct healthcare, and unemployment costs (8).

Pharmacological interventions, including antipsychotic medications, constitute the cornerstone of disease management in both the acute and maintenance treatment of BP-I (9, 10); however, upwards of 50% of patients living with bipolar disorder demonstrate suboptimal treatment adherence (11–13). Compared with adherent patients, those with poor adherence experience greater disease severity and functional impairment (12), an increased risk of relapse, hospitalization, and suicide attempts, and greater healthcare resource utilization (HCRU) (14). Reduced adherence is also associated with higher direct and indirect medical costs (14–16).

Adherence to antipsychotics may be improved with long-acting injectable (LAI) formulations (17). Studies utilizing real-world datasets have indicated that, compared with a daily oral antipsychotic, treatment with an LAI antipsychotic is associated with improved adherence and lower rates of discontinuation in patients diagnosed with BP-I or bipolar disorder more generally (18, 19). Further, use of LAI versus oral antipsychotics is associated with fewer relapses or hospitalizations in populations of patients living with bipolar disorder (20, 21).

A ripiprazole once-monthly (AOM) and aripiprazole 2-month ready-to-use (Ari 2MRTU; approved in 2023) are LAIs containing aripiprazole monohydrate that are approved for the maintenance monotherapy treatment of adults diagnosed with BP-I in the US (22–24) (indications vary by region and clinicians should refer to their local prescribing information). While AOM and Ari 2MRTU demonstrate similar safety, efficacy, and pharmacokinetic profiles, Ari 2MRTU offers less frequent dosing (once every 56 days) and greater dosing flexibility (administration within 2 weeks before or after the scheduled injection date, depending on region and local prescribing information), while maintaining stable plasma concentrations and ensuring control of BP-I symptoms over the full 2-month dosing interval (23, 25–27).

Among people living with BP-I, transitioning from a daily oral antipsychotic or AOM to Ari 2MRTU may lead to improvements in treatment adherence (e.g. due to the reduced dosing frequency). As improved adherence would be expected to reduce the risk of relapse, in turn this may lead to reductions in HCRU and medical costs due to the reduced demand on the healthcare system. However, this has not yet been evaluated in real-world clinical practice, reflecting the relatively recent approval of Ari 2MRTU and its so-far limited real-world availability. The present retrospective study used a pre-post design to assess treatment adherence, HCRU, and cost outcomes over 6 months of follow-up in a large US claims cohort of adults diagnosed with BP-I who transitioned to Ari 2MRTU from either oral antipsychotics or AOM.

## 2 Materials and methods

### 2.1 Study objectives

The primary objective of this study was to assess treatment adherence, HCRU, and costs in patients diagnosed with BP-I who transitioned from daily oral antipsychotics to Ari 2MRTU. An exploratory objective was to evaluate treatment adherence, HCRU, and costs in patients diagnosed with BP-I who transitioned from AOM to Ari 2MRTU.

### 2.2 Study design and claims dataset

**Figure 1:**
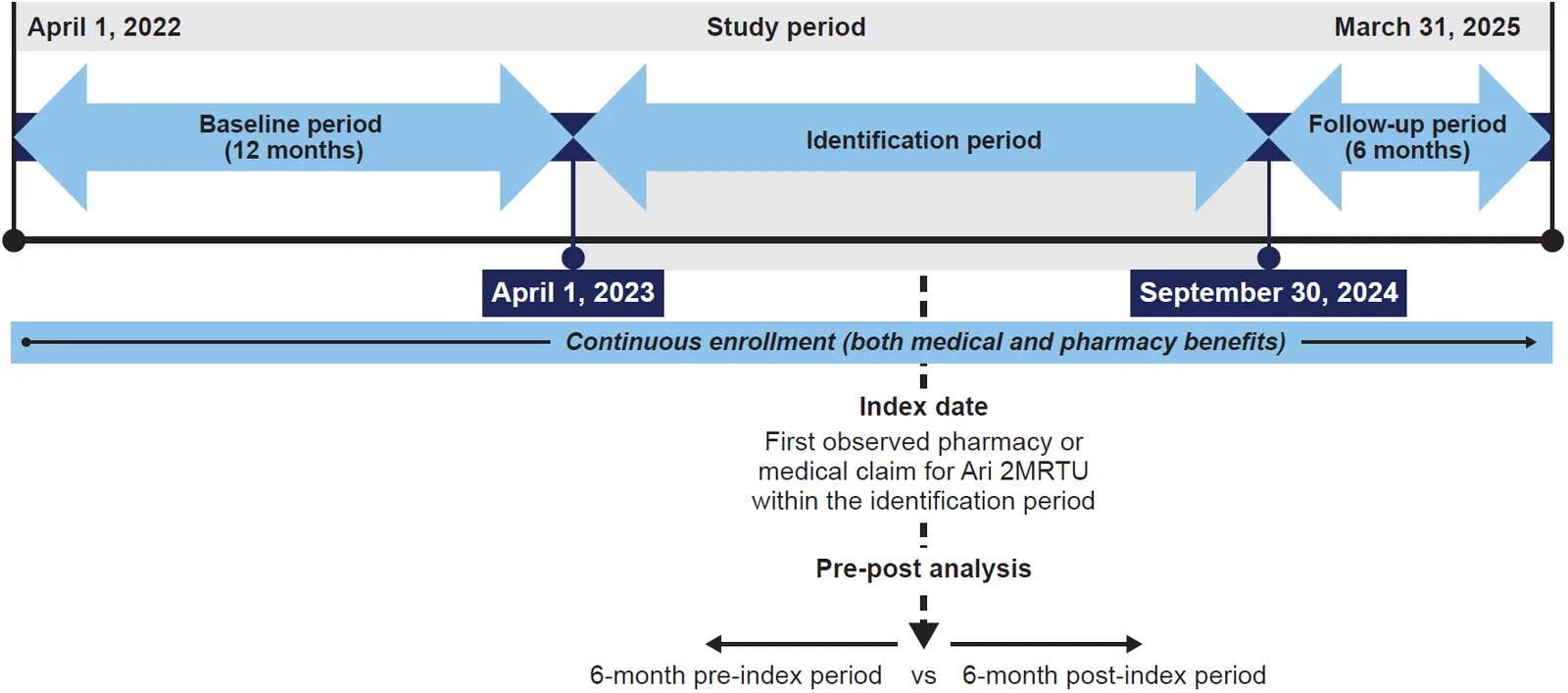
Study design. Ari 2MRTU, aripiprazole 2-month ready-to-use.

This retrospective, noninterventional cohort study analyzed data from the Kythera Labs closed-claims dataset relating to commercially insured and Medicaid-enrolled patients in the US. The design of the study is shown in **Figure 1**; in brief, the study period extended from April 1, 2022 to March 31, 2025. Patients were indexed on the date of their first observed pharmacy or medical claim for Ari 2MRTU occurring within an identification period spanning April 1, 2023, to September 30, 2024. Patients must have been continuously enrolled in the database for 12 months prior to the index date (baseline period) and 6 months after the index date (follow-up period), with no interruption in insurance coverage (where an interruption in coverage was defined as a gap of >30 days). Information regarding the Kythera Labs claims dataset, including data sources and coverage, patient demographics, insurance type, diagnostic and procedural coding, medication data, and longitudinal linkage, has been reported previously (28, 29). The closed-claims subset used for the current analysis included 172 million commercially insured members and 30 million Medicaid enrollees.

### 2.3 Eligibility criteria

Patients were eligible for inclusion in the current analysis if they had at least one pharmacy or medical claim for either the 960 mg or 720 mg dose of Ari 2MRTU during the identification period, captured using National Drug Codes (NDCs) (pharmacy claims) and/or Healthcare Common Procedure Coding System codes (medical claims). All included patients met the following additional criteria: (i) a diagnosis of BP-I identified by either at least one inpatient claim or at least two outpatient claims with an International Classification of Diseases, 10^th^ Revision, Clinical Modification (ICD-10-CM) diagnosis code of F31* occurring at least 30 days apart during the baseline period; (ii) age of at least 18 years on the index date; and (iii) continuous health plan enrollment with medical and pharmacy benefits during the baseline and follow-up periods. To ensure all eligible patients were included, patients were identified using all diagnosis codes in the F31* family, including F31.8 (other bipolar disorders, including bipolar II disorder) and F31.9 (bipolar unspecified). Therefore, the diagnosis code alone does not give a definitive identification of BP-I in claims data. However, as Ari2MRTU is indicated for maintenance monotherapy treatment of bipolar I disorder in adults in the US, initiation of Ari2MRTU among patients with bipolar disorder claims served as a claims-based proxy for treatment in a BP-I maintenance context. An advantage of using F31* to identify patients (rather than specific subcodes) was that if a patient changed diagnosis during the study period (e.g. from unspecified to specified type) it did not affect their classification or inclusion as long as they were contributing qualifying claims throughout. Patients were excluded from the study if they had a dual diagnosis of bipolar disorder (type not specified; any code in the F31* family) and schizophrenia (any code in the F20* family) on the same day during the study period (baseline or follow-up), to improve specificity of the BP-I cohort. Patients were also excluded if they had an insurance coverage gap of >30 days during the 12 months before and 6 months after the index date or if they were prescribed any LAI antipsychotic other than the study drug during the study period.

### 2.4 Study populations

Two mutually exclusive cohorts were established according to prior antipsychotic treatment. The first cohort comprised patients diagnosed with BP-I transitioning from oral antipsychotics to Ari 2MRTU (i.e., those with at least one pharmacy claim for any oral antipsychotic during the baseline period). Oral antipsychotics included atypical and typical preparations marketed in the US (including those without a labelled indication for BP-I), identified using NDCs encompassing branded and generic products from multiple manufacturers across a range of package configurations. The second cohort comprised patients diagnosed with BP-I transitioning from AOM to Ari 2MRTU (i.e., those with at least one pharmacy or medical claim for AOM during the baseline period).

Demographic and clinical characteristics were captured during the 12-month baseline period and were summarized for each cohort using descriptive statistics. Socio-demographic variables of interest — obtained from relevant fields within the Kythera Labs commercial claims dataset — included sex, age, payer type, and region. Additionally, a previously established summary measure of socioeconomic status (SES) for each US ZIP code was applied, incorporating income, education, and occupation (30), using data from the 2021 US Census 5-year estimates; patients were sorted and categorized into low, medium, or high SES terciles based on their summary scores. Prescriber specialty was determined by linking the National Provider Identifier for each prescriber to their primary taxonomy code recorded in the National Plan and Provider Enumeration System. In this manner, prescribers were categorized as psychiatric specialists or primary care providers, with physician assistants separately categorized as a distinct prescriber group. Baseline mental health and systemic comorbidities were identified using relevant ICD-10-CM codes, while comorbidity burden for the 12-month baseline period was assessed using established tools: the Chronic Disease Score (31); the Elixhauser comorbidity measure (32); and the updated version of the Charlson Comorbidity Index, which uses ICD-10-CM codes and weighted medical condition categories to capture both inpatient and outpatient diagnoses (33, 34).

### 2.5 Study outcomes

Study outcomes were assessed using a pre-post design that compared medication adherence, all-cause and BP-I-related HCRU, and all-cause medical costs in the 6-month periods prior to and following the index date. Medication adherence was analyzed in three ways: (i) proportion of days covered (PDC), defined as the ratio of the number of days in the period “covered” by the medication to the total number of days within that period (reflecting treatment coverage over time); (ii) medication possession ratio (MPR), defined as the ratio of the number of days’ supply of medication to the total number of days within that period (reflecting medication supplied over time); and (iii) the percentage of patients considered adherent within each period, defined as an MPR of ≥ 0.8. In the case of PDC, the days of supply for AOM and Ari 2MRTU was defined as the minimum of the time between injections and the labeled dosing interval; this reflects that the days’ supply field is often missing or unreliable for LAI antipsychotics in medical and pharmacy claims datasets, respectively. (Related to this, in the context of LAI antipsychotics, PDC is considered a more stable measure of adherence than MPR, which is sensitive to variations in the days’ supply field (35).) HCRU was assessed for both all-cause and BP-I–related outcomes, encompassing hospital length of stay (LOS) as well as the number of inpatient hospitalizations and outpatient and emergency department (ED) visits, reported as the mean value per patient and as the proportion of patients with any utilization. BP-I–related encounters were those with a primary ICD-10-CM diagnosis code of F31*. Costs included all-cause direct medical costs and inpatient, outpatient, ED, and total medical expenditures, adjusted to 2024 US dollars using the medical care component of the Consumer Price Index. Patients were not required to have continuous Ari 2MRTU possession for inclusion or continued follow-up, to avoid the survivor/adherer bias that would arise if follow-up were censored or restricted to patients who remained continuously on Ari 2MRTU.

Continuous variables were summarized as means with standard deviations, while categorical variables were presented as counts and percentages. Between-period differences were evaluated using two-sided tests for within-cohort comparisons (paired *t*-tests for continuous outcomes, chi-square tests for categorical outcomes) at a significance level of α = 0.05. All analyses were performed using Apache Spark 3.5.2 (Scala 2.12) and PySpark version 4.0.0.

## 3 Results

### 3.1 Patient population

**Figure 2:**
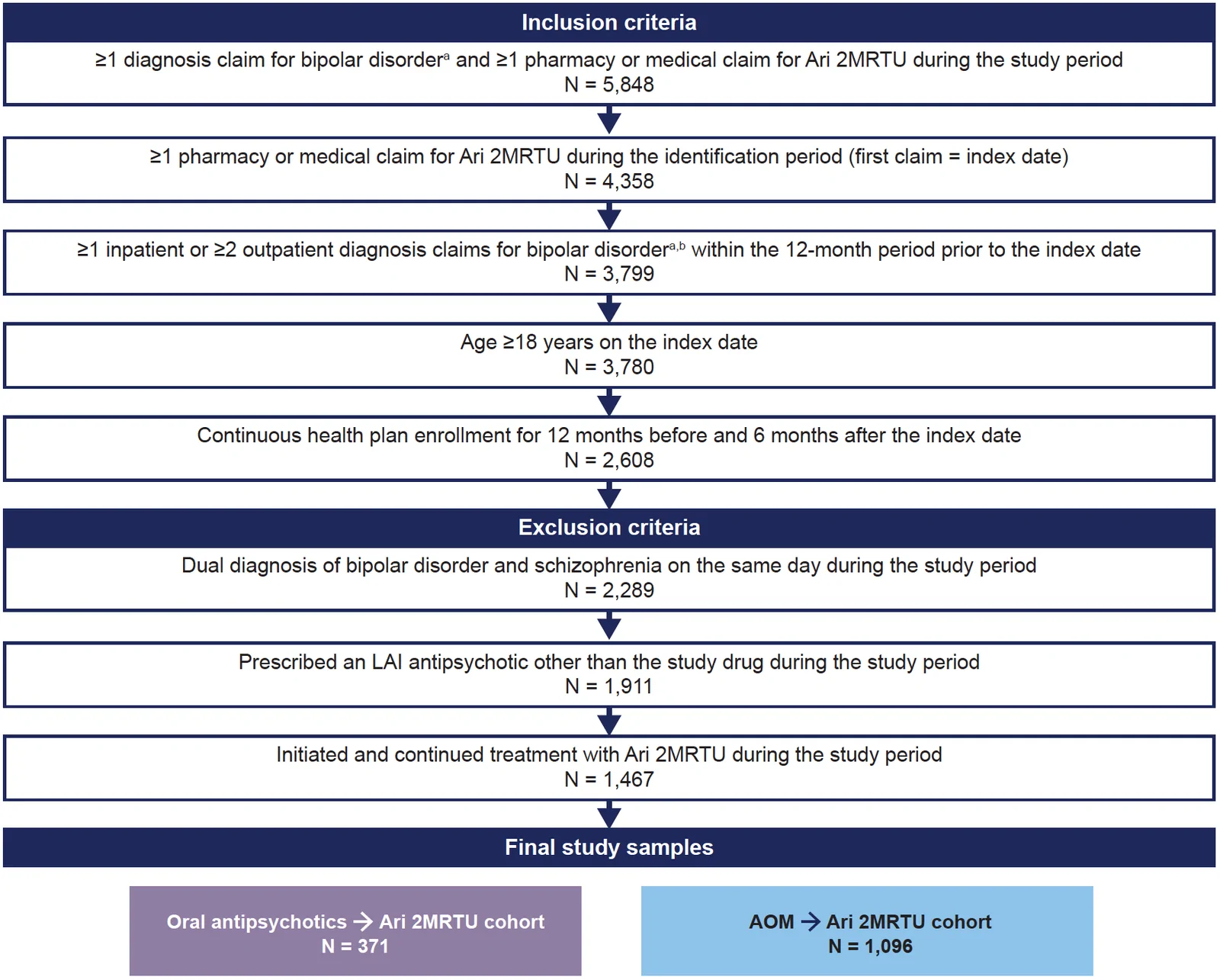
Selection of patient cohorts. ^a^International Classification of Diseases, 10^th^ Revision, Clinical Modification diagnosis code of F31*; ^b^occurring ≥30 days apart. AOM, aripiprazole once-monthly; Ari 2MRTU, aripiprazole 2-month ready-to-use; LAI, long-acting injectable.

After applying the inclusion and exclusion criteria, the final study sample comprised 371 patients in the oral antipsychotics to Ari 2MRTU cohort and 1,096 patients in the AOM to Ari 2MRTU cohort (**Figure 2**). Across both cohorts, 83.8% of all Ari 2MRTU prescriptions during the follow-up (including the index date) were for a 960 mg dose, and 16.2% were for a 720 mg dose. The baseline demographic and clinical characteristics of patients who transitioned from oral antipsychotics to Ari 2MRTU, and from AOM to Ari 2MRTU, were generally similar, although numerical between-cohort differences were noted for some characteristics, including index year, prescriber specialty, and rates of certain mental health comorbidities (**Table 1**). Mean (SD) age was 38.5 (13.5) years and 39.6 (13.3) years in the oral antipsychotic and AOM cohorts, respectively, with the largest percentage of patients (26.1% and 27.9%, respectively) in the age group of 25–34 years. Most patients were commercially insured and under the care of psychiatric specialists. Mental and systemic health comorbidities were common.

**Table 1:** Baseline demographic and clinical characteristics for patients who transitioned from oral antipsychotics or AOM to Ari 2MRTU.

Characteristics	Oral antipsychotic to Ari 2MRTU (N = 371)	AOM to Ari 2MRTU (N = 1,096)
Age, mean (SD)	38.46 (13.5)	39.60 (13.3)
Age groups, n (%)		
Age group: 18–24 years	72 (19.4)	148 (13.5)
Age group: 25–34 years	97 (26.1)	306 (27.9)
Age group: 35–44 years	84 (22.6)	259 (23.6)
Age group: 45–54 years	67 (18.1)	210 (19.2)
Age group: 55–64 years	36 (9.7)	134 (12.2)
Age group: ≥ 65 years	15 (4.0)	39 (3.6)
Sex, n (%)		
Female	192 (51.8)	543 (49.5)
Male	123 (33.2)	383 (34.9)
Unknown	56 (15.1)	170 (15.5)
Payer type, n (%)		
Commercial	278 (74.9)	836 (76.3)
Medicaid	93 (25.1)	260 (23.7)
Index year, n (%)		
2023	94 (25.3)	454 (41.4)
2024	277 (74.7)	642 (58.6)
US geographic region, n (%)		
Northeast	53 (14.3)	130 (11.9)
Midwest	89 (24.0)	337 (30.7)
South	131 (35.3)	397 (36.2)
West	94 (25.3)	222 (20.3)
Socioeconomic status, n (%)		
Low tercile	122 (32.9)	397 (36.2)
Middle tercile	121 (32.6)	370 (33.8)
High tercile	121 (32.6)	310 (28.3)
Prescriber specialty, n (%)		
Psychiatric specialists	235 (63.3)	585 (53.4)
Primary care providers	53 (14.3)	120 (10.9)
Physician assistants	18 (4.9)	69 (6.3)
Other^a^	65 (17.5)	322 (29.4)
Comorbidity scores		
CCI score, mean (SD)	0.7 (1.2)	0.7 (1.2)
CCI score categories, n (%)		
0	233 (62.8)	684 (62.4)
1	80 (21.6)	232 (21.2)
≥ 2	58 (15.6)	180 (16.4)
Chronic Disease Score, mean (SD)	3.6 (2.9)	3.1 (2.8)
Elixhauser Comorbidity Index Score, mean (SD)	5.2 (3.1)	4.3 (3.1)
Mental health comorbidities [ICD-10 CM code], n (%)		
Any mental health comorbidity^b^	306 (82.5)	793 (72.4)
Substance use disorders [F10-F19]	218 (58.8)	501 (45.7)
Major depressive disorder [F32.0-F32.5, F32.9, F33.x]	127 (34.2)	272 (24.8)
Anxiety disorders [F40-41]	206 (55.5)	503 (45.9)
Post-traumatic stress disorder [F43.1]	108 (29.1)	258 (23.5)
Systemic health comorbidities [ICD-10 CM code], n (%)		
Any systemic health comorbidity^c^	217 (58.5)	621 (56.7)
Diabetes [E10-E14]	82 (22.1)	278 (25.4)
Obesity [E66]	60 (16.2)	183 (16.7)
Hypertension [I10-I15]	116 (31.3)	287 (26.2)
Dyslipidemia [E78]	70 (18.9)	259 (23.6)
Cardiovascular disease [I25.10]	8 (2.2)	24 (2.2)
Metabolic syndrome [E88.81]	0 (0.0)	8 (0.7)
Sleeping disorders [G47]	86 (23.2)	239 (21.8)
Chronic obstructive pulmonary disease [J44]	26 (7.0)	86 (7.8)
Chronic kidney disease [N18]	15 (4.0)	41 (3.7)
Epilepsy [G40]	26 (7.0)	53 (4.8)
Hypothyroidism [E03]	31 (8.4)	97 (8.9)

^a^ Includes providers who were not classified as psychiatric specialists, primary care providers, or physician assistants based on their taxonomy codes.

^b^ Includes anxiety disorders, major depressive disorder, substance use disorders, and posttraumatic stress disorder.

^c^ Includes obesity, diabetes, hypertension, dyslipidemia, cardiovascular disease, chronic obstructive pulmonary disease, chronic kidney disease, epilepsy, hypothyroidism, metabolic syndrome, and sleep disorders.

AOM, aripiprazole once-monthly; Ari 2MRTU, aripiprazole 2-month ready-to-use; CCI, Charlson Comorbidity Index; SD, standard deviation.

### 3.2 Adherence, HCRU, and all-cause medical costs in patients 6 months pre- and post-transition from oral antipsychotics to Ari 2MRTU

#### 3.2.1 Adherence

**Figure 3:**
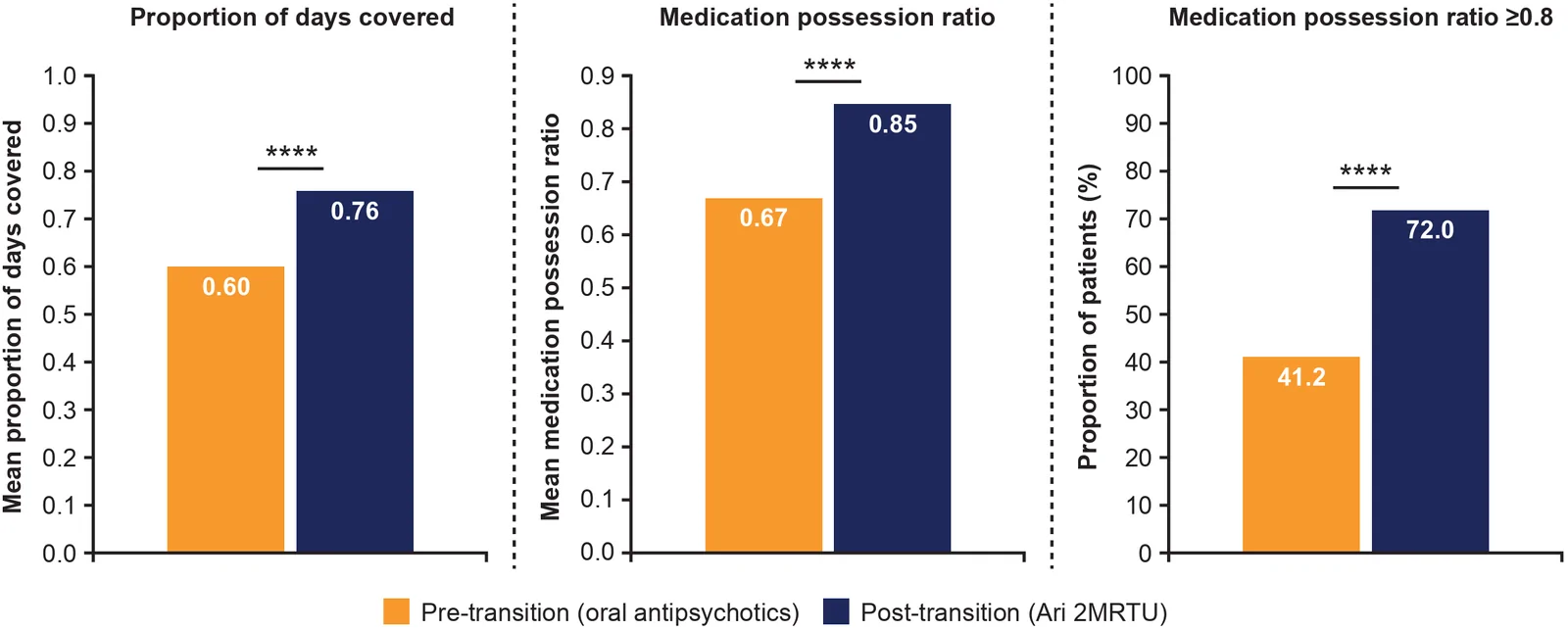
Adherence 6 months pre- and post-transition from oral antipsychotics to Ari 2MRTU in patients diagnosed with BP-I. ****P < 0.0001 for between-period difference. Ari 2MRTU, aripiprazole 2-month ready-to-use; BP-I, bipolar I disorder.

In the 6-month period following a transition from oral antipsychotics to Ari 2MRTU, mean PDC increased from 0.60 to 0.76 (26.2% relative increase), mean MPR increased from 0.67 to 0.85 (26.8% relative increase), and the proportion of patients classified as adherent (MPR ≥ 0.8) increased from 41.2% to 72.0% (74.5% relative increase) (all *P* < 0.0001) (**Figure 3**).

#### 3.2.2 All-cause and BP-I–related HCRU (mean value per patient)

**Figure 4:**
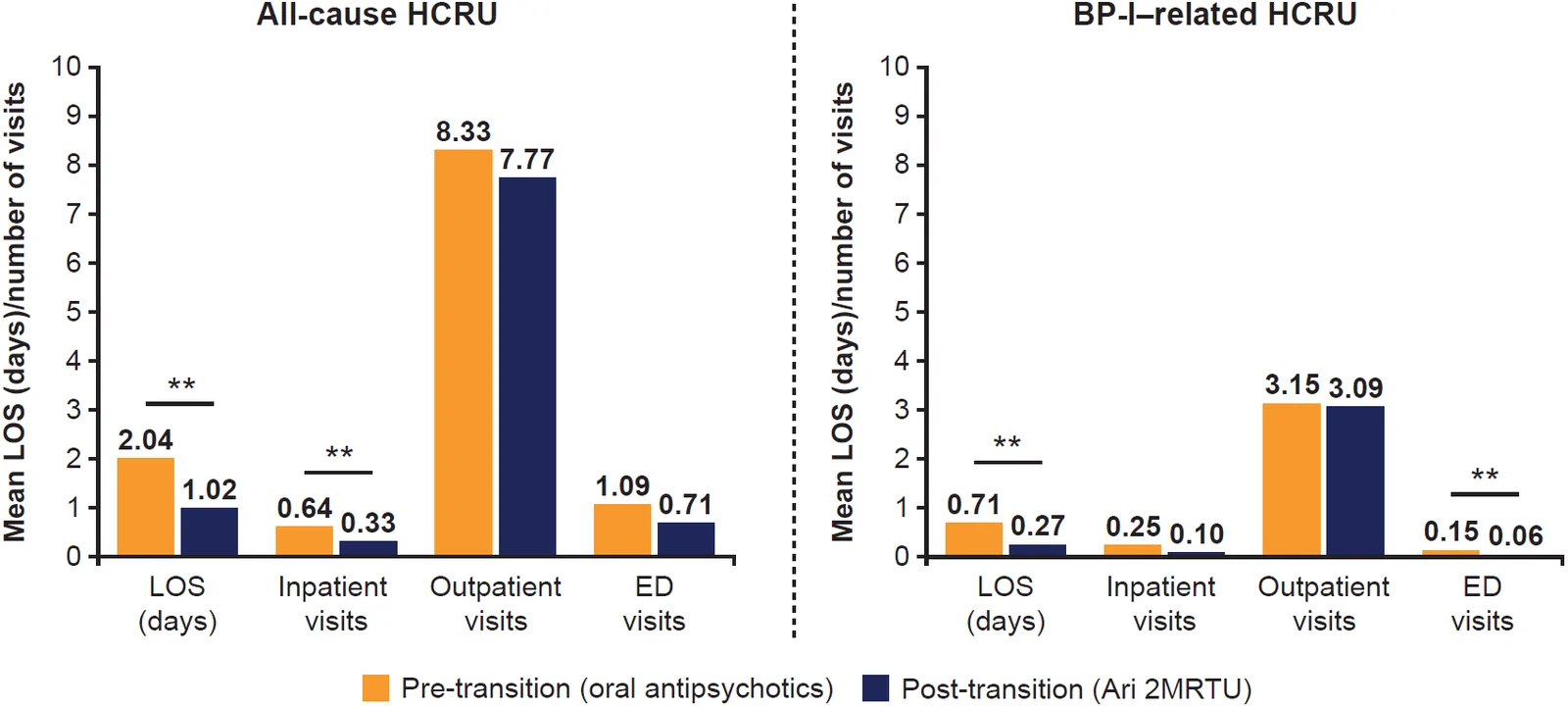
All-cause and BP-I–related HCRU 6 months pre- and post-transition from oral antipsychotics to Ari 2MRTU in patients diagnosed with BP-I. **P < 0.01 for between-period difference. Ari 2MRTU, aripiprazole 2-month ready-to-use; BP-I, bipolar I disorder; ED, emergency department; HCRU, healthcare resource utilization; LOS, length of stay.

Regarding all-cause HCRU, there was a decrease from the pre-transition period to the 6-month post-transition period in the mean number of inpatient visits (0.64 vs 0.33; *P* = 0.0073) and the mean inpatient LOS (2.04 vs 1.02 days; *P* = 0.0010) (**Figure 4**). Regarding BP-I–related HCRU, there was a decrease from the pre-transition period to the 6-month post-transition period in the mean inpatient LOS (0.71 vs 0.27 days; *P* = 0.0028) and the mean number of ED visits (0.15 vs 0.06; *P* = 0.0061) (**Figure 4**).

#### 3.2.3 All-cause and BP-I–related HCRU (proportion of patients with any utilization)

Regarding all-cause HCRU, a decrease from the pre- to post-transition period was observed in the proportions of patients with at least one inpatient stay (26.4% vs 14.3%; *P* = 0.0037) and at least one ED visit (36.4% vs 24.3%; *P* = 0.0110). These changes were accompanied by a decrease in the proportion of patients with at least one outpatient visit (89.2% vs 77.6%; *P* = 0.0027). For BP-I–related HCRU, decreases from the pre- to post-transition period were also observed for the proportions of patients with at least one inpatient stay (11.9% vs 5.4%; *P* = 0.0265) and at least one ED visit (9.4% vs 3.2%; *P* = 0.0142). Again, these changes were accompanied by a decrease in the proportion of patients with at least one outpatient visit (62.0% vs 42.3%; *P* = 0.0001).

#### 3.2.4 All-cause medical costs (per patient per year)

**Figure 5:**
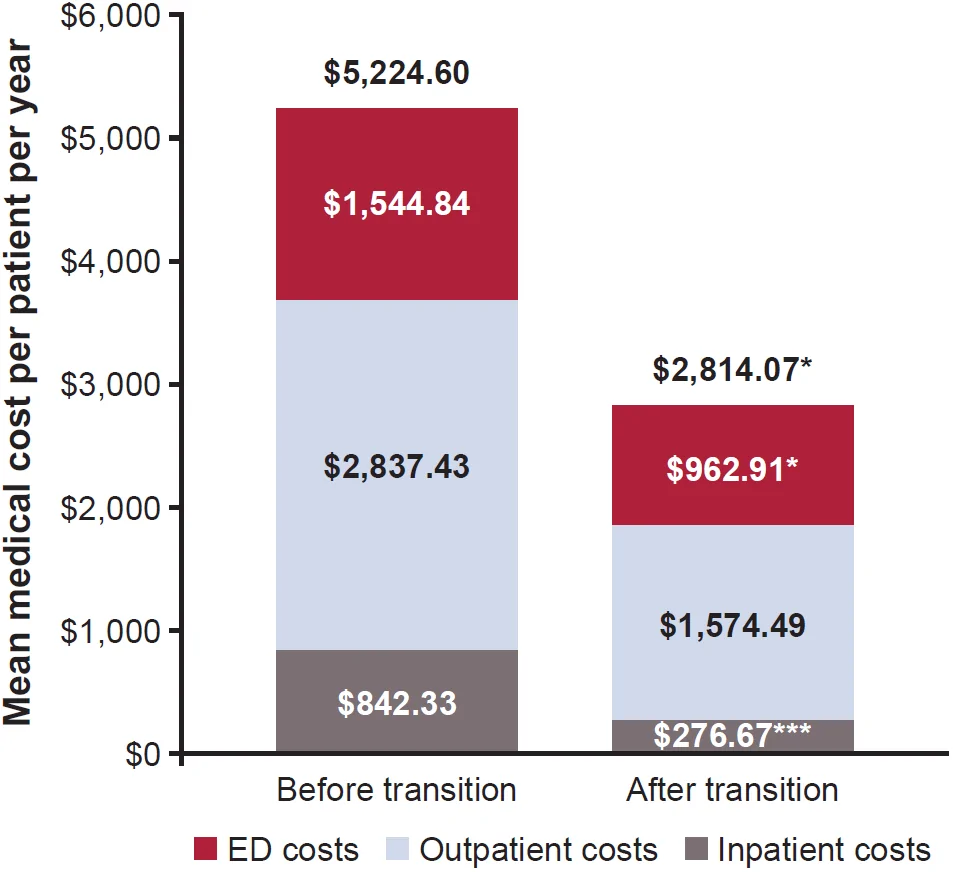
All-cause medical costs 6 months pre- and post-transition from oral antipsychotics to Ari 2MRTU in patients diagnosed with BP-I. *P < 0.05; ***P < 0.001 for between-period difference. Ari 2MRTU, aripiprazole 2-month ready-to-use; BP-I, bipolar I disorder; ED, emergency department.

Total all-cause medical costs decreased following the transition to Ari 2MRTU, from $5,224.60 in the pre-transition period to $2,814.07 in the 6-month post-transition period (*P* = 0.0154). This reflected a $565.66 decrease in mean inpatient costs (*P* = 0.0001), a $1,262.94 decrease in mean outpatient costs, and a $581.93 decrease in ED costs (*P* = 0.0387) (**Figure 5**).

### 3.3 Adherence, HCRU, and all-cause medical costs in patients 6 months pre- and post-transition from AOM to Ari 2MRTU

#### 3.3.1 Adherence

**Figure 6:**
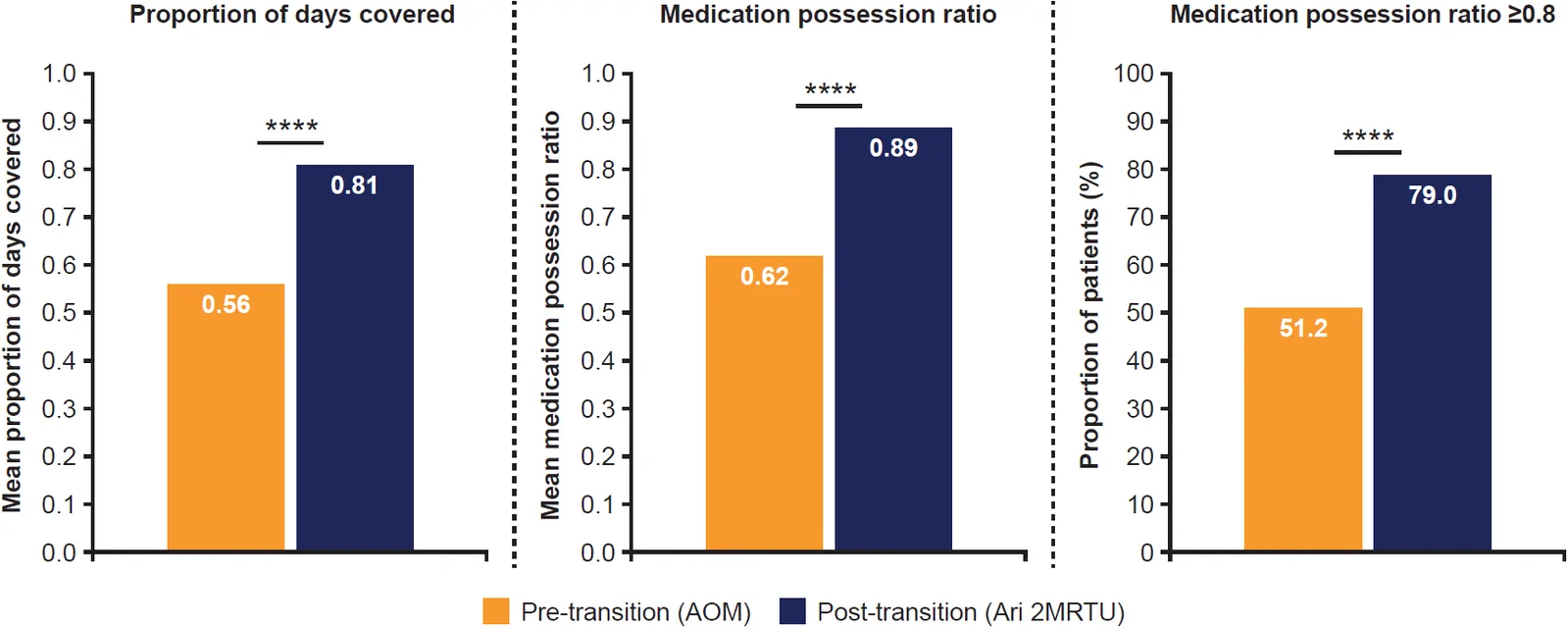
Adherence 6 months pre- and post-transition from AOM to Ari 2MRTU in patients diagnosed with BP-I. ****P < 0.0001 for between-period difference. AOM, aripiprazole once-monthly; Ari 2MRTU, aripiprazole 2-month ready-to-use; BP-I, bipolar I disorder.

In the 6-month period following a transition from AOM to Ari 2MRTU, mean PDC increased from 0.56 to 0.81 (45.3% relative increase), mean MPR increased from 0.62 to 0.89 (43.7% relative increase), and the proportion of patients with an MPR ≥ 0.8 increased from 51.2% to 79.0% (54.4% relative increase) (all *P* < 0.0001; **Figure 6**).

#### 3.3.2 All-cause and BP-I–related HCRU (mean value per patient)

**Figure 7:**
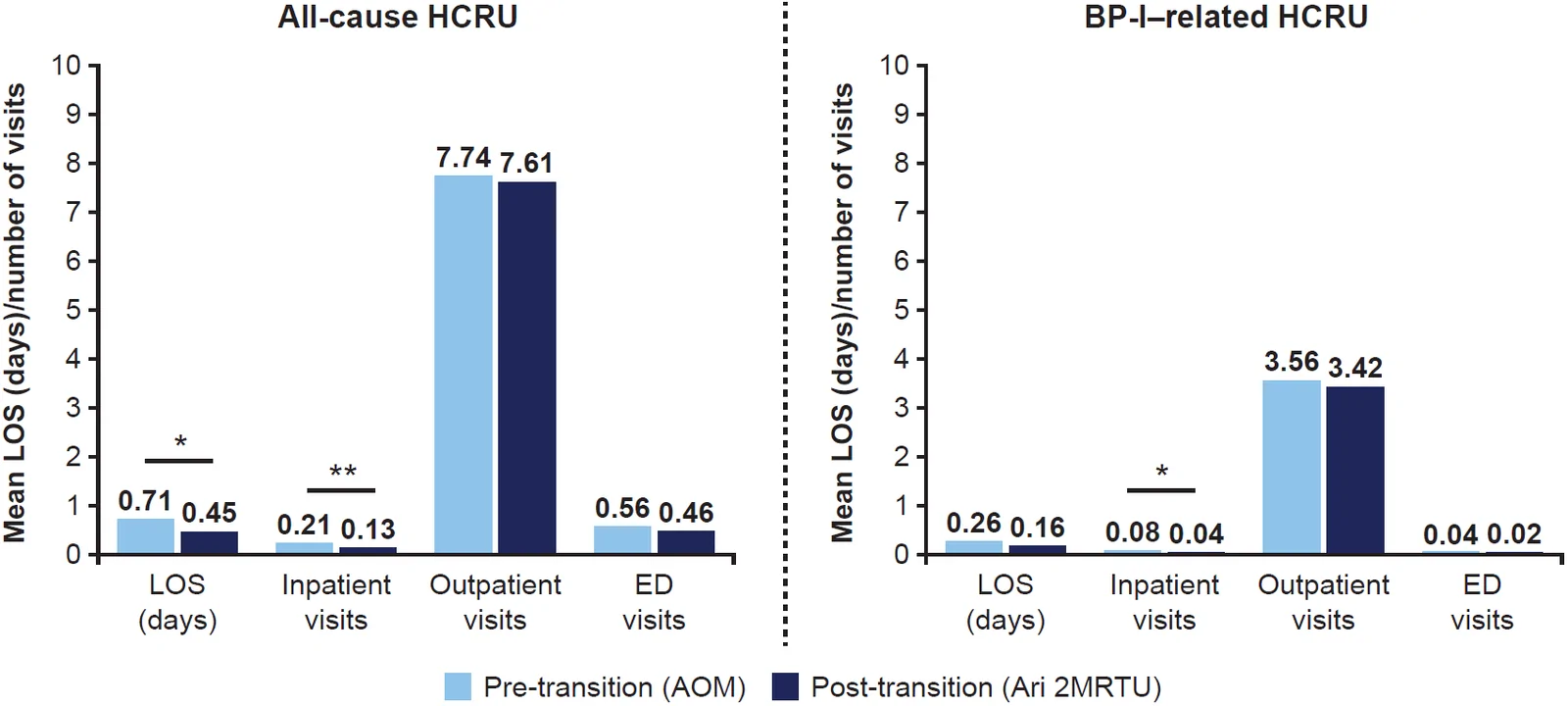
All-cause and BP-I–related HCRU 6 months pre- and post-transition from AOM to Ari 2MRTU in patients diagnosed with BP-I. *P < 0.05; **P < 0.01 for between-period difference. AOM, aripiprazole once-monthly; Ari 2MRTU, aripiprazole 2-month ready-to-use; BP-I, bipolar I disorder; ED, emergency department; HCRU, healthcare resource utilization; LOS, length of stay.

Improvements were observed for most all-cause HCRU outcomes in the 6-month post-transition compared with the pre-transition period, including fewer inpatient visits per patient (0.21 vs 0.13; *P* = 0.0097) and a shorter inpatient LOS (0.71 vs 0.45 days; *P* = 0.0246) (**Figure 7**). Regarding BP-I–related HCRU, there was a notable decrease in the number of inpatient visits per patient post-transition to Ari 2MRTU (0.08 vs 0.04; *P* = 0.0160) (**Figure 7**).

#### 3.3.3 All-cause and BP-I–related HCRU (proportion of patients with any utilization)

Regarding all-cause HCRU, there was a numerical decrease from the pre- to post-transition period in the proportions of patients with at least one inpatient stay (10.2% vs 7.0%; *P* = 0.0597) and at least one ED visit (20.0% vs 17.3%; *P* = 0.2609). In contrast, the proportion of patients with at least one outpatient visit increased significantly after transition (69.9% vs 80.3%; *P* = 0.0001). Regarding BP-I–related HCRU, the proportion of patients with at least one inpatient stay numerically decreased from 4.9% pre-transition to 2.7% post-transition (*P* = 0.0590).

#### 3.3.4 All-cause medical costs (per patient per year)

**Figure 8:**
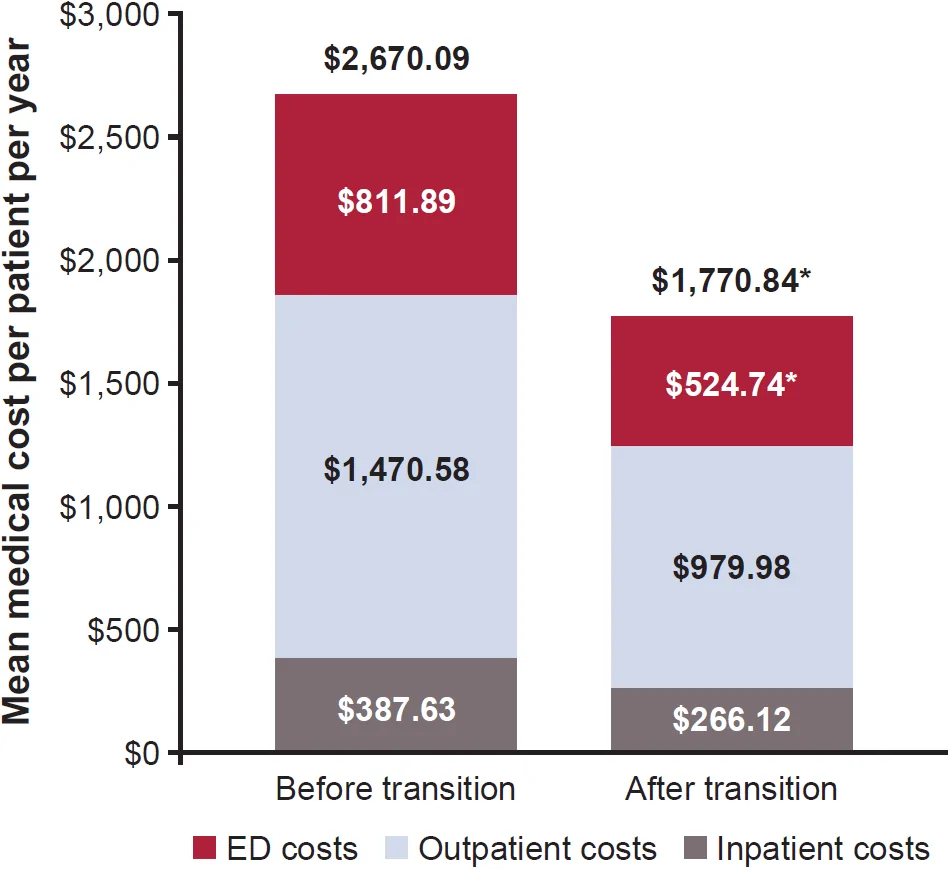
All-cause medical costs 6 months pre- and post-transition from AOM to Ari 2MRTU in patients diagnosed with BP-I. *P < 0.05 for between-period difference. AOM, aripiprazole once-monthly; Ari 2MRTU, aripiprazole 2-month ready-to-use; BP-I, bipolar I disorder; ED, emergency department.

There was a significant reduction in all-cause total medical costs following a transition to Ari 2MRTU, from $2,670.09 in the pre-transition period to $1,770.84 in the 6-month post-transition period (*P* = 0.0139). This reflected a $121.51 decrease in mean inpatient costs, a $490.60 decrease in mean outpatient costs, and a $287.15 decrease in ED costs (*P* = 0.0148) (**Figure 8**).

## 4 Discussion

This retrospective claims-based analysis suggests that transitioning adults diagnosed with BP-I from oral antipsychotics or AOM to Ari 2MRTU was associated with improvements in medication adherence and reductions in certain HCRU measures and all-cause medical costs over 6 months of follow-up.

The observed improvements in adherence after a transition from oral antipsychotics to Ari 2MRTU mirror results from previous real-world studies of LAI antipsychotics. Data from previous claims database analyses in patients diagnosed with BP-I (or bipolar disorder more generally) indicate that LAI antipsychotic treatment is associated with improved adherence and a lower rate of discontinuation compared with patients receiving oral antipsychotics (18, 19). Furthermore, results from the present study suggest that transitioning from AOM to Ari 2MRTU is also associated with significant improvements in adherence. This finding may stem from the extended dosing interval of Ari 2MRTU and an associated reduced treatment burden. Data from patients living with schizophrenia indicate that LAI antipsychotics with dosing intervals of longer than 1 month may be preferred due to improved convenience, reduced treatment burden, and the potential to mitigate stigma (36–39). Similar findings were derived from a qualitative interview study that explored LAI antipsychotic preferences among people living with BP-I and caregivers (40, 41). Respondents noted that one of the most important features in the selection of an LAI antipsychotic was the frequency of administration, with an ideal treatment being one that was dosed less often than once monthly (40, 41). Respondents viewed a hypothetical once-every-2-months LAI antipsychotic positively, due to increased convenience (less time thinking about treatment), reduced personal impact (fewer injections, less responsibility, improved relationships, and more freedom), feeling more “normal”, and reduced impact on caregivers (40, 41).

Mean PDC, mean MPR, and the proportion of patients with an MPR of ≥ 0.8 in the current study were generally similar in the pre-transition period in the oral antipsychotic to Ari 2MRTU cohort and the AOM to Ari 2MRTU cohort, suggesting similar baseline adherence in the two cohorts. While the study was designed to evaluate within- rather than between-cohort differences, this observation is noteworthy since patients transitioning from AOM might reasonably be expected to have higher baseline adherence than those transitioning from an oral antipsychotic (e.g., because of a reduced reliance on daily tablet taking). That said, LAIs do not completely eliminate non-adherence, and delayed or missed injections do occur in routine clinical practice. While it is not possible to determine the reasons for treatment switching in the current study, it is plausible that suboptimal adherence to AOM contributed to the clinical decision to transition to Ari 2MRTU, at least in some patients. If this was the case, treatment adherence in this cohort may not be representative of all patients receiving AOM. Regardless, similarly high levels of adherence were observed in both cohorts after patients transitioned to Ari 2MRTU.

In addition to an improvement in treatment adherence, the current study also identified reductions in certain hospitalization-related HCRU measures (i.e., inpatient hospitalizations and ED visits) with Ari 2MRTU. Results from the oral antipsychotics to Ari 2MRTU cohort align with prior literature on LAI antipsychotic treatment for bipolar disorder, including BP-I, showing lower risks and rates of psychiatric and all-cause rehospitalization, as well as reductions in hospitalization frequency and duration with LAI versus oral antipsychotics (20, 21, 42–45). Of note, a retrospective analysis demonstrated that BP-I–related 30- and 60-day rehospitalization rates were significantly lower in patients treated with LAI versus oral antipsychotics in a US hospital database study (42). Similarly, a systematic review and random-effects meta-analysis of observational studies in individuals diagnosed with bipolar disorder demonstrated a significantly lower risk of psychiatric hospitalizations and a significantly lower number of hospitalization days and hospitalizations due to mania (20). Lastly, in a retrospective mirror-image study in patients with bipolar disorder, AOM (n=25) was associated with substantially lower hazards of psychiatric hospitalization, hospitalization days, psychiatric ED visits, and mood episode recurrence, while paliperidone palmitate once-monthly (n=17) was associated with substantially lower hazards of hospitalization and mood episode recurrence (21).

Previously, there was a lack of data comparing HCRU outcomes in patients diagnosed with BP-I following a transition from a once-monthly LAI to an LAI with a longer dosing interval. The current study suggests that patients using a once-monthly maintenance LAI antipsychotic (AOM) can potentially achieve improvements in select hospitalization-related HCRU measures with a transition to an LAI with a longer dosing interval (Ari 2MRTU). These findings align with evidence suggesting reductions in HCRU with LAI antipsychotic dosing intervals that extend beyond 1 month in a schizophrenia setting (46, 47).

The current study showed mixed results for changes in the proportion of patients with at least one all-cause outpatient visit from the pre- to post-index period. While a significant reduction was observed for this outcome in the oral antipsychotic to Ari 2MRTU cohort, a significant increase occurred among patients who transitioned from AOM to Ari 2MRTU (although the mean number of outpatient visits per patient stayed roughly the same). The observed increase in patients with any outpatient utilization in the AOM to Ari 2MRTU cohort may relate to more frequent short-term follow-up visits to monitor tolerability and response with a new LAI formulation. Clinicians may also use initiation of a new LAI antipsychotic as an opportunity for broader patient engagement and a more holistic care model involving regular contact outside of injection visits (48), including assessment of comorbidities and psychosocial needs. In this context, the observed increase in patients who accessed outpatient services may reflect improved engagement, monitoring, and continuity of care rather than excess utilization, and could partly explain reductions in inpatient and ED encounters. Collectively, changes in outpatient service use following a transition to Ari 2MRTU should be interpreted cautiously since neither an increase nor a decrease provides definitive evidence of better or worse treatment adherence or effectiveness.

Consistent with reductions in certain HCRU measures, a transition to Ari 2MRTU was also associated with notable decreases in all-cause medical costs across both cohorts, primarily driven by lower inpatient and/or ED expenditures. These findings add to those of a budget impact analysis that showed that the introduction of Ari 2MRTU as a maintenance treatment for BP-I in the US was expected to be cost neutral with respect to payer budgets (49).

This study should be interpreted considering several important limitations. Retrospective study designs are limited by their reliance on data that were not specifically collected for research, which may lead to missing information (50). Related to this, claims datasets are primarily designed for billing and reimbursement, meaning that they lack detailed clinical information that would provide greater context (28) (e.g., reasons for non-adherence), and may be subject to misclassification (51, 52). The retrospective nature of the data and the pre-post study design, without a comparator or control group, limits causal inference, as observed changes may reflect factors other than the intervention. For instance, improvements observed after transitioning to Ari 2MRTU may, in part, reflect secular trend effects or regression to the mean (53, 54). More specifically, if patients were transitioned to Ari 2MRTU during a period of instability, some improvement may have occurred over time irrespective of treatment. This may be especially relevant given the study’s inclusion criterion of at least one pharmacy/medical claim for an oral antipsychotic or AOM during the 12-month baseline period, which establishes treatment exposure but does not provide insight into medication adherence or clinical status immediately before transition. Accordingly, improvements in HCRU and costs may partly reflect recovery from a period of clinical instability. Similarly, improvements may partly reflect changes in concomitant pharmacotherapy such as mood stabilizers, antidepressants, or other antipsychotics, which may form part of a broader treatment regimen in patients living with BP-I, but which were not considered in the current analysis. Additionally, if prescribers preferentially select patients who are motivated and adherent for transition, observed improvements post-transition may partly reflect this, rather than being attributable to the medication alone. The relatively short 6-month follow-up period limits the ability to capture longer-term outcomes such as relapse, rehospitalization, and durable persistence. Short-term gains in adherence may also be inflated by a “honeymoon effect” (55), where additional support, motivation, and provider contact temporarily boosts adherence after switching. Claims data cannot confirm whether medications were actually administered by a healthcare professional as prescribed, and adherence measures may be affected in the event of a delayed or missed dose (56, 57). While both PDC and MPR were used to mitigate this bias, these proxies still risk overestimating true medication-taking behavior (35), particularly in the absence of clinical verification. This reflects that measures of adherence were developed for oral medications and do not consider the unique characteristics of LAI formulations (e.g., no ability to stockpile medication, potential delay between fill date and administration date) (35). Therefore, the reported Ari 2MRTU post-index adherence may be inflated, and treatment gaps may be underestimated, and comparisons between baseline oral or AOM adherence and post-index Ari 2MRTU adherence should be interpreted cautiously. The study population largely consisted of commercially insured patients, with Medicaid-enrolled participants underrepresented and Medicare and uninsured patients not represented. This limits the generalizability of the results, which may not be applicable to older adults, those with a higher comorbidity burden, or those in lower socioeconomic strata. Similarly, results may not necessarily be generalizable to regions outside of the US. Further, the small sample size, particularly in the oral antipsychotic cohort, may have reduced statistical power (58). Lastly, because statistical comparisons were based on unadjusted tests and did not account for within-person correlation or the non-normal distributions typical of HCRU outcomes, results should be interpreted descriptively. Despite these limitations, administrative claims data provide important real-world perspectives on treatment patterns and outcomes. Patient records included in the Kythera Labs database are generally complete and missing data are rare (59). To ensure accuracy in situations where data are incomplete, patients are benchmarked according to their enrollment status and characteristics and cross-referenced with alternative data sources (59).

## 5 Conclusion

In this retrospective, pre-post analysis of adults diagnosed with BP-I in clinical practice, transitioning from oral antipsychotics or AOM to Ari 2MRTU was associated with significant improvements in treatment adherence over 6 months of follow-up, as well as reductions in select HCRU measures, including fewer ED visits, fewer inpatient admissions, and shorter hospital stays, and reduced all-cause medical costs. Future studies with longer follow-up periods and more detailed clinical data that permit multivariable analyses are needed to confirm these results.

## Data Availability

The data analyzed in this study is subject to the following licenses/restrictions: The raw dataset on which this study is based is available through a commercial data licensing agreement with Kythera Labs. Requests to access these datasets should be directed to https://www.kytheralabs.com/contact-us-kythera-labs.
